# Cooking for disability: a pilot study on nutritional interventions for mental health support in adults with autism spectrum disorder

**DOI:** 10.3389/fpsyt.2025.1608033

**Published:** 2025-08-19

**Authors:** Johanna Maria Catharina Blom, Ciro Ruggerini, Franco Caroli, Carla Ferreri, Annalisa Masi, Veronica Rivi, Pierfrancesco Sarti, Mauro Rebecchi, Chiara Arletti

**Affiliations:** ^1^ Dept. of Biomedical, Metabolic and Neural Sciences, University of Modena and Reggio Emilia, Modena, Italy; ^2^ Association for the Inclusion of People with Severe Disabilities, Modena, Italy; ^3^ Consiglio Nazionale delle Ricerche (CNR)-Institute for Organic Synthesis and Phtoreactivity (ISOF), Bologna, Italy; ^4^ Consiglio Nazionale delle Ricerche (CNR)-Institute of Crystallography (IC), Montelibretti, Italy; ^5^ Dept. of Life Sciences, University of Modena and Reggio Emilia, Modena, Italy; ^6^ Public Institutions for Personal Care Services Charitas, Azienda pubblica dei servizi alla persona (ASP) Charitas di Modena, Italy

**Keywords:** nutritional psychiatry, research domain criteria, neuroinflammation, omega-3/omega-6 fatty acid profiles, behavioral phenotyping

## Abstract

**Background:**

Autism Spectrum Disorder (ASD) is a complex neurodevelopmental condition often accompanied by chronic inflammation and metabolic dysfunction, which are increasingly recognized as key contributors to symptom severity and behavioral challenges. Despite these insights, targeted nutritional interventions in adults with severe ASD remain understudied.

**Aim:**

This pilot study investigated whether a precision, biomarker-guided dietary intervention could improve both behavioral symptoms and underlying biological dysregulations in adults with Level 3 ASD.

**Methods:**

Seven adults with severe ASD residing at the ASP Charitas residential facility in Modena (Italy) participated in a 12-month dietary intervention aimed at reducing inflammation, optimizing fatty acid profiles, and supporting metabolic health. Behavioral assessments—including CARS-2-ST, DASH-II, ABC, and SSP—and biological markers such as IL-6, fecal calprotectin, vitamin D, HbA1c, and erythrocyte lipid profiles were collected at baseline, 6 months, and 12 months.

**Results:**

Baseline assessments confirmed severe ASD symptoms, psychiatric comorbidities, sensory processing abnormalities, systemic and intestinal inflammation, metabolic impairment, and a pro-inflammatory lipid profile. Following the intervention, significant reductions were observed in core autism symptoms (CARS-2-ST, ABC), alongside decreased frequency and severity of behavioral symptoms (DASH-II). Sensory processing (SSP) stabilized or modestly improved. Biochemical markers shifted toward normalization, including increased vitamin D, improved insulin sensitivity (HbA1c), and reduced inflammatory markers (IL-6, fecal calprotectin). Lipidomic profiling revealed elevated anti-inflammatory omega-3 fatty acids (DHA, EPA) and a decreased omega-6/omega-3 ratio. Correlational analyses linked these biological changes to behavioral improvements, suggesting a mechanistic connection.

**Conclusions:**

This pilot study suggests that a targeted dietary intervention, based on biological markers reflective of neurobiological dysfunctions, may offer a promising complementary approach to managing ASD symptoms in adults. The findings indicate that dietary modifications can lead to improvements in both biological and behavioral markers, but further research is needed to refine personalized nutritional strategies for individuals with ASD.

## 1 Introduction

Adults with Autism Spectrum Disorder (ASD) frequently present a complex array of co-occurring psychiatric symptoms (1, 2)—such as anxiety, mood dysregulation, and severe behavioral challenges—that profoundly impair quality of life, heighten caregiver burden, and often require sustained long-term residential care (3–5). Left untreated, co-occurring psychiatric and behavioral conditions in individuals with ASD can have profoundly detrimental effects (6) on both the individuals and their families (7). Currently, psychopharmacological treatments for ASD in these settings are commonly prescribed off-label (6, 8, 9), reflecting a limited availability of approved medications tailored to this population. This landscape poses significant challenges for healthcare providers, who must balance the growing demand for clinical support with the complexities and uncertainties inherent in off-label prescribing (7, 10). In addition to the psychiatric and behavioral challenges, individuals with ASD also face higher rates of physical health concerns, including a significantly greater prevalence of being overweight, obesity, and constipation compared to neurotypical peers (11). For instance, studies report that 19% of individuals with ASD are overweight compared to 16% in the general population, and 30.4% are obese versus 23.6% among neurotypical individuals (11). In recent years, research has increasingly focused on the relationship between ASD and eating behaviors, identifying common patterns such as food selectivity, behavioral rigidity, specific meal rituals, and neophobia (12). These behaviors often result in highly restricted diets and the elimination of entire food groups, which can further exacerbate nutritional imbalances (13, 14). For instance, lipidomic imbalances and chronic low-grade inflammation have been observed in subsets of individuals with ASD, and these biological alterations may be responsive to specific dietary modifications (13). Given these multifaceted challenges and the complexities of management of adults with ASD, nutritional psychiatry (15) emerges as a promising complementary approach (16). By targeting underlying biological mechanisms such as inflammation, oxidative stress, and metabolic imbalances, precision-based dietary interventions have the potential to support mental health and behavioral regulation alongside standard treatments (11, 16–19). This integrative strategy may be particularly valuable for adults with severe ASD (9), a population often underserved by conventional care models (20, 21). Moreover, the evolving landscape of ASD research increasingly emphasizes the need to move beyond traditional diagnostic categories toward a deeper understanding of the underlying biology (12). The National Institute of Mental Health’s Research Domain Criteria (RDoC) framework embodies this paradigm shift (22, 23) by encouraging investigation into underlying neurobiological mechanisms (22), such as mitochondrial dysfunction (23), and chronic low-grade inflammation (24). This biologically grounded perspective opens the door for novel, non-pharmacological interventions that directly target these physiological processes (25). Our pilot study introduces an innovative, biomarker-informed approach to nutritional intervention for adults with severe ASD. Conducted in collaboration with caregivers and clinical staff at ASP Charitas, the study implemented a 6-month dietary intervention. Starting from baseline assessments of inflammatory markers, lipidomic data, and oxidative stress indicators, we designed personalized meal plans and cooking routines adapted to the residential context.

The central aim of the study was to assess the feasibility and preliminary impact of such a nutritional approach on both biological and behavioral outcomes (26). Specifically, we investigated whether dietary interventions could lead to measurable changes in neurobiological markers associated with mental health and whether these changes would be accompanied by improvements in mood, anxiety, and maladaptive behaviors. This dual focus on both objective physiological indicators and observable behavioral outcomes allowed for a more comprehensive assessment of the intervention’s potential (27). For this pilot study, several key objectives are advanced. First, it investigates a feasible, non-pharmacological intervention that can be integrated into residential care routines. Second, it puts into practice the concepts of precision psychiatry and the RDoC framework within a real-world setting, providing a practical model for individualized treatment based on biological markers rather than diagnosis alone. Third, it aims to empower adults with ASD and their caregivers by highlighting nutrition and meal preparation as accessible tools for enhancing self-regulation, overall health, and mental well-being. Ultimately, this pilot seeks to generate early, practice-driven evidence supporting biologically informed interventions in autism. By demonstrating both feasibility and promising outcomes in everyday care environments, it establishes a foundation for larger trials and advances the creation of holistic, person-centered care models for adults with ASD.

## 2 Methods

### 2.1 Participants, diagnostic criteria, and inclusion and exclusion criteria

The study sample comprised seven individuals residing in a single living unit at the ASP Charitas residential facility in Modena, Italy. All participants met the criteria for ASD requiring “very substantial support” (Level 3), as defined by the Diagnostic and Statistical Manual of Mental Disorders, Fifth Edition, Text Revision (DSM-5-TR). Diagnostic confirmation was based on comprehensive clinical evaluations and supported by scores from the Childhood Autism Rating Scale, Second Edition – Standard Version (CARS-2-ST), administered by licensed clinicians with expertise in neurodevelopmental disorders. Participants were eligible for inclusion if they had a confirmed diagnosis of Level 3 ASD, presented with significant behavioral or psychiatric comorbidities that rendered family-based care unfeasible, and had resided at the facility for a minimum of three years to ensure environmental stability during the intervention. Exclusion criteria included the presence of unstable medical conditions, known food allergies that would interfere with adherence to the dietary protocol, or recent changes in psychotropic medications (within 60 days prior to study initiation). Detailed demographic and clinical information—including age, sex, and duration of residency—is provided in the Results section.

### 2.2 Biological indices selection and rationale

In our study, we selected specific biological indices to assess potential neurobiological dysfunctions in individuals with ASD. These indices and their rationales, supported by current literature, are as follows:

- Anti-Transglutaminase IgA (tTG Ab): Anti-Transglutaminase IgA (tTG Ab) was measured to assess potential gluten-related immune reactivity. As gluten-related immune responses, even in the absence of celiac disease, have been reported in some individuals with ASD, tTG Ab was included in this study as it may contribute to gastrointestinal or behavioral symptoms (28).
- Fecal calprotectin: Calprotectin is a protein found in neutrophils, macrophages, and monocytes that is a marker for gastrointestinal inflammation (29). Given the high prevalence of gastrointestinal issues in individuals with ASD (30), assessing fecal calprotectin provides insight into gut inflammation and its potential impact on behavior.
- Glycated Hemoglobin (HbA1c) reflects average blood glucose levels over the past two to three months and is used to assess insulin resistance (31), which, in turn, has been linked to cognitive impairments and may contribute to neurodevelopmental disorders (31).
- Interleukin-6 (IL-6): IL-6 is a multifunctional cytokine involved in immune responses associated with oxidative stress (32). Elevated IL-6 levels have been implicated in neuroinflammation and have been observed in individuals with ASD (33), suggesting a role in the disorder’s pathophysiology.
- 25-Hydroxy Vitamin D (25(OH)D): Vitamin D is a steroid hormone involved in brain enzymatic regulation and neuronal protection against free radicals and inflammation (34), whose deficits have been associated with various neuropsychiatric disorders, including ASD, highlighting the importance of monitoring its levels (35).
- Long-chain polyunsaturated fatty acids (PUFAs): These compounds, particularly from the omega-6 and omega-3 families, play a key role in regulating neuronal function, inflammation, and membrane dynamics (36). In the context of neurodevelopmental disorders such as ASD, imbalances in these fatty acids—especially an elevated omega-6/omega-3 ratio—have been associated with a pro-inflammatory state, impaired membrane fluidity, and altered synaptic signaling (37, 38). In particular, arachidonic acid (AA, omega-6) serves as a precursor for pro-inflammatory eicosanoids (39), whereas docosahexaenoic acid (DHA, omega-3) and eicosapentaenoic acid (EPA) are precursors to specialized pro-resolving compounds (40–42), which are involved in neuronal protection, resolution of inflammation, and neuroplasticity (43, 44). Therefore, profiling the membrane fatty acid composition provides insight into the neuroinflammatory status and metabolic resilience of individuals with ASD. Since PUFA composition is largely influenced by dietary intake (45) this approach allows for both the identification of nutritional imbalances and the monitoring of response to dietary interventions (46).

### 2.3 Membrane lipidomics analysis

To assess the lipidomic status of participants, we analyzed the fatty acid composition of cell membrane phospholipids, with a specific focus on mature red blood cells (RBCs) (47). Due to their approximately 120-day lifespan and incorporation of fatty acids into membrane phospholipids during erythropoiesis (48), RBCs provide a stable and integrative measure of fatty acid availability over time, allowing for evaluation of dietary fatty acid intake and endogenous membrane remodeling processes associated with cellular growth and turnover (40–42). The membrane lipidomic analysis of RBCs was performed using an analytical protocol certified to comply with ISO 17025 requirements, ensuring the reliability and repeatability of the results. In particular, the analytical process involves the transformation of membrane phospholipids isolated from mature red blood cells into fatty acid methyl esters (FAME, then analysed by gas chromatography), using a mild alkaline procedure that is very respectful of the PUFA residues, particularly those of plasmalogens, known to be sensitive to acidic and oxidative conditions (49). The fatty acid composition of mature RBC membrane phospholipids was obtained from blood samples (about 2 mL) collected in vacutainer tubes containing ethylenediaminetetraacetic acid (EDTA). Samples were shipped to the ISO 17025-certified Lipidomic Laboratory (LAB #01622, Lipinutragen srl, Bologna) at a controlled temperature and, upon arrival, underwent quality control to verify the absence of hemolysis. During blood processing, before lipid extraction and the conversion to fatty acid methyl esters (FAMEs), the automated protocol included selecting mature RBCs, as previously reported (50). Briefly, the whole blood in EDTA was centrifuged (4000 revolutions per minute (rpm) for 5 minutes at 4°C), and the mature cell fraction was isolated based on the higher density of aged cells (50), with the process controlled using a cell counter (Scepter 2.0 with Scepter™ Software Pro, EMD Millipore, Darmstadt, Germany). All subsequent steps were automated, including cell lysis, isolation of membrane pellets, phospholipid extraction from pellets using the Bligh and Dyer method (51), transesterification to FAMEs by treatment with a potassium hydroxide (KOH)/methyl alcohol (MeOH) solution (0.5 mol/L) for 10 minutes at room temperature, and extraction using hexane (2 mL). FAMEs were analysed via capillary column gas chromatography (GC). GC analysis was performed on the Agilent 6850 Network GC System (Agilent, USA), equipped with a fused silica capillary column Agilent DB23 (60 m × 0.25 mm × 0.25 μm) and a flame ionisation detector. Achieving optimal separation of all fatty acids and their geometrical and positional isomers, identification, and calibration were performed by comparison to commercially available standards and a library of trans isomers of MUFAs and PUFAs. The quantity of each FA was calculated as a percentage of the total FA content, then converted to a percentage of the total fatty acid quantity (quantitative relative percentage), with over 97% of the GC peaks identified.

### 2.4 Behavioral measures

To establish a comprehensive baseline profile of each participant’s functional abilities, sensory processing characteristics, and psychiatric symptoms, a battery of standardized instruments was administered. Core features of autism and overall functioning were assessed using the Childhood Autism Rating Scale, Second Edition – Standard Version (CARS-2-ST) (52). This structured observational tool, appropriate for assessing individuals aged 2 years and older, evaluates 15 domains including verbal and nonverbal communication, emotional response, body use, adaptation to change, and sensory sensitivities. Scores yield a severity index of autism spectrum symptoms, ranging from minimal to severe, and support clinical confirmation of ASD diagnosis. The CARS-2-ST was selected for its focus on behavioural characteristics that remain salient across the lifespan, particularly in adults with high support needs (53). Unlike many adult-specific tools that rely on verbal fluency or self-report, the CARS-2-ST is based on direct observation and caregiver input, making it especially appropriate for individuals with limited expressive language or intellectual disability (54). To assess sensory processing patterns in daily life, the Short Sensory Profile (SSP) was completed by caregivers. This questionnaire evaluates behavioral responses to sensory stimuli across multiple domains (e.g., tactile, auditory, proprioceptive), identifying patterns of hypersensitivity or hyposensitivity that may influence behavior, interaction, and daily functioning (55). Both the CARS-2-ST and the SSP were administered at baseline and the end of the intervention. Psychiatric symptoms and behavioral disturbances were systematically evaluated at all three time points using two validated instruments specifically suited for individuals with severe neurodevelopmental disorders:

- The Diagnostic Assessment for the Severely Handicapped – Second Edition (DASH-II), a validated informant-based checklist specifically designed for individuals with intellectual and developmental disabilities, that measures the presence, frequency, and severity of psychiatric symptoms such as anxiety, depression, and psychotic features (56).
- The Aberrant Behavior Checklist (ABC), which assesses maladaptive and disruptive behaviors, including irritability, hyperactivity, and social withdrawal (57).

### 2.5 Dietary modifications

The dietary regimen for study participants was systematically modified to incorporate anti-inflammatory and neuroprotective nutritional strategies aimed at improving overall metabolic and behavioral health. The primary objectives were to reduce inflammatory dietary components, optimize macronutrient distribution, and enhance the balance of essential fatty acids, particularly omega-3 and omega-6 polyunsaturated fatty acids (PUFAs).

Fat content in daily meals was calculated by analyzing each recipe’s ingredients based on official nutritional databases (CREA and BDA). Meals were designed to contain total fat levels below 23.3 ± 3.8%, with saturated fatty acids (SFA) limited to 16.6 ± 2.6%. The omega-6 to omega-3 ratio was maintained around 3.2 ± 2.4, and the saturated fat to monounsaturated fat (SFA/MUFA) ratio was controlled at approximately 0.28 ± 0.9. Biscuits provided as snacks contained less than 5% total fat.

Key dietary modifications included the following:

- Reduction in overall carbohydrate intake, emphasizing low-gluten, whole-grain sources such as whole-grain rice and durum wheat flour (Type 1).
- Replacement of sucrose with xylitol, a sugar alcohol with a lower glycemic index and calorie content, to reduce pro-inflammatory sweeteners.
- Daily inclusion of oilseeds, specifically walnuts and flaxseeds, to boost intake of polyunsaturated fatty acids, particularly alpha-linolenic acid (ALA), supported by a daily dose of flaxseed oil.
- Increased consumption of fish to three servings per week to supply long-chain omega-3 fatty acids essential for neuronal function.
- Introduction of two weekly legume-based meals (chickpeas, lentils, beans, peas) to provide plant-based protein and fiber.
- Reduction of potato servings to help regulate glycemic response.
- Exclusive use of whole-grain and semi-whole-grain breads made from durum wheat or semi-integrated wheat flour.
- Daily consumption of fresh, seasonal fruits and vegetables, with vegetables served cooked or raw twice daily.
- Limiting meat consumption to five meals per week, balancing white and red meats to control saturated fat intake while ensuring adequate protein.
- Increasing egg-based meals to three to four times per week to diversify protein sources.
- Inclusion of two weekly meals featuring fresh cheeses such as ricotta and unsweetened yogurt to provide probiotics and calcium.
- Modification of breakfast to include 85% dark chocolate, chestnut honey, plain yogurt, and ricotta cheese to supply polyphenols, balanced fats, and probiotics.
- Two customized snack recipes—a biscuit (“Orecchie di Napoleone”) and crackers—were developed with attention to taste and texture to promote palatability and adherence throughout the day.

The dietary changes were actively implemented by kitchen staff, educators, and the participants’ families. One author (FC) supervised adherence to the nutritional guidelines throughout the intervention. Under the guidance of a Michelin-starred chef, special attention was paid to food texture, shape, and color to enhance sensory appeal and acceptability for the participants.

Additionally, water intake was actively monitored throughout the day by the center’s caregiving personnel to ensure participants maintained adequate hydration levels consistent with clinical guidelines and individual needs. However, the exact quantities of water consumed were not systematically recorded. This approach was adopted considering the practical constraints of the care environment and the primary focus on ensuring sufficient hydration rather than precise volumetric measurement. Although quantitative tracking of water intake can provide valuable data, in this context, continuous qualitative monitoring by experienced staff was deemed sufficient to prevent dehydration and support overall participant well-being without imposing additional burdens on participants or caregivers.

### 2.6 Study timeline

The study was structured over three distinct time points to carefully monitor both behavioral and neurobiological changes while minimizing confounds related to external supplementation.

- At T0 (Baseline Assessment), comprehensive evaluations were conducted to establish each participant’s initial status; these evaluations included the collection of neurobiological markers, both direct and indirect behavioral assessments, and a detailed characterization of baseline dietary intake. This initial phase was critical for capturing the participants’ natural physiological and nutritional profiles prior to any interventions.
- At T1 (after 6 months of dietary intervention), the same array of biological and behavioral indices was reassessed to determine the effects of the modified diet alone. This phase allowed us to observe changes attributable to the dietary modifications, without the confounding influence of supplementary nutrients.
- At T2 (after an additional 6 months combining continued dietary modifications with targeted supplementation), participants underwent a final assessment. During this period, supplementation was introduced only for those with documented deficiencies or persistent imbalances, as determined by predefined biochemical thresholds and observed trends in individual nutrient status.

This stepwise approach was designed to ensure that the initial improvements could be ascribed solely to dietary changes. It also provided an opportunity to tailor subsequent nutritional support in an evidence-based and personalized manner, thereby enhancing both the scientific rigor and the clinical relevance of the intervention. Overall, this sequential design allowed for a clear differentiation between the effects of natural dietary intake, the isolated impact of dietary modification, and the combined influence of diet plus targeted supplementation on neurobiological and behavioral outcomes in adults with severe ASD.

### 2.7 Data analysis

The distribution of all variables was assessed for normality using both the Shapiro-Wilk and Kolmogorov-Smirnov tests, supplemented by visual inspection of Q-Q plots. Several key variables deviated significantly from normality (p < 0.05), justifying the use of non-parametric methods where appropriate. Spearman’s rank-order correlation was employed to compute the correlation matrix, given its robustness to non-normal distributions and outliers, and its suitability for small sample sizes. Statistical significance of correlations was evaluated at a 95% confidence level. The resulting correlation matrix was visualized using the corrplot package in R; non-significant correlations were left blank to enhance interpretability. For behavioral and psychological measures administered at all three time points (T0, T1, T2), repeated-measures ANOVAs were performed. When the assumption of sphericity was violated (as assessed by Mauchly’s test), the Greenhouse-Geisser correction was applied. Significant main effects were followed up with Dunnett’s *post-hoc* tests to compare each time point to baseline (T0). The same analytic approach was applied to biological indices and lipidomic data collected at the same time points. For questionnaires administered only at baseline (T0) and post-intervention (T2), paired-sample t-tests were used to evaluate pre–post differences. A significance threshold was set at p < 0.05. However, given the small sample size, statistical power was limited, increasing the risk of both Type I and Type II errors. This limitation inherently constrains the reliability and generalizability of inferential statistics. Accordingly, the primary aim of the study was exploratory: to assess the feasibility of the nutritional intervention, identify potential trends, and generate preliminary data for future, larger-scale trials. In this context, we prioritized descriptive statistics and applied inferential tests only where appropriate and interpretable, while clearly acknowledging their limitations. Substantial inter-individual variability and the absence of a control group further complicate formal hypothesis testing. To avoid overstating findings, we present observed changes over time transparently and cautiously, consistent with the exploratory nature of the study and in line with methodological rigor. All analyses were conducted using RStudio (version 2025.05.1) and all the Figures were generated using GraphPad Prism 8.

## 3 Results

### 3.1 Participant demographics

The final study sample consisted of seven individuals (6 males, 1 female) residing in one of the living units at the ASP Charitas residential facility in Modena, Italy. Participants ranged in age from 19 to 48 years, with a mean age of 28.8 years (SD = 9.6). All individuals had long-term placement histories at the facility, reflecting the severity of their clinical profiles and the inability to maintain care within the family context. The length of residence varied from 4 to 24 years, with a mean duration of 9.7 years (SD = 6.5). All participants had been diagnosed with ASD requiring very substantial support (Level 3), as defined by the DSM-5-TR, and exhibited significant co-occurring psychiatric conditions or behavioral challenges at the time of the study.

### 3.2 Baseline characteristics: biological and behavioral profiles

At the onset of the study (Time 0), participants exhibited a high degree of variability in biological and behavioral measures, reflecting a complex interplay between metabolic, inflammatory, and neurobehavioral factors (**Table 1**).

**Table 1:** Individual baseline scores for behavioral assessments and biological markers across the seven participants.

Participant		1	2	3	4	5	6	7
Test	Cut-off/Range	Test scoring						
Behavioral indices at baseline								
**CARS-2 - Total score**	>30	*53.5	*42.5	*37	*45.5	*39	*47.5	*44
**DASH-II (severity + frequency)**	No specific cut-off for both severity and frequency	113	63	108	94	101	85	83
- Impulse and control		32	13	30	21	21	22	17
- Cognitive deterioration		13	9	10	10	10	8	3
- Anxiety		8	3	0	2	4	1	9
- Depression		17	16	17	14	14	14	4
- Mania		11	5	11	11	11	10	7
- Autism		12	6	8	14	16	12	12
- Schiophrenia		2	0	2	1	2	2	4
- Stereotypies		8	2	8	10	10	10	14
- Self-injuries behaviours		8	3	3	2	4	3	7
- Elimination disorders		2	0	2	2	0	0	3
- Eating disorders		0	2	12	3	6	0	0
- Sleep disorders		0	0	2	2	2	3	1
- Sexual disorders		0	4	3	2	1	0	2
**SSP - Total score**	<154	*146	156	*150	174	*150	*131	*143
- Tactile sensitivity	<29	*28	*27	*28	35	*25	*24	*11
- Taste and olfactory sensitivity	<14	16	20	20	20	20	*11	20
- Sensitivity to movement	<12	13	13	13	15	15	15	*3
- Hyporesponsiveness	<26	*19	26	*11	*22	*17	*13	30
- Auditory filtering	<22	*20	24	23	30	*20	*16	25
- Low energy/ weakness	<25	30	*23	30	30	30	30	30
- Visual/auditory sensitivity	<18	20	23	25	22	23	22	24
**ABC - Total score**	0-174	70	39	72	34	69	77	72
- Irritability	0-45	30	11	20	8	24	21	19
- Lethargy - Social withdrawal	0-48	5	14	4	9	10	9	26
- Stereotypies	0-21	11	0	14	9	8	17	14
- Hyperactivity	0-48	18	12	32	8	22	30	13
- Inappropriate speech	0-12	6	2	2	0	5	0	0
Biological markers at baseline								
Fecal calprotectin	<50µg/g negative 50-120 borderline >120 positive	15	3.7	*614	*295	39	42	*219
Glycated Hemoglobin (HbA1c)	>39 mmol/mol pre-diabetes	32	33	33	26	32	33	36
Creatine Kinase (CK)	20- 200 U/L	69	38	135	*259	100	*372	99
C-Reactive Protein	> 3 mg/L high 1–3: moderate <1: low risk	0.5	0.9	0.5	0.5	0.5	0.5	0.5
Interleukin-6	> 6.4pg/ml positive	*68.5	*13	*37.8	*7.9	*11.1	0.7	5.5
Vitamin D	<20ng/ml deficit <10 severe deficit	*16.9	*7.2	22.6	*6.1	20.2	*17	*28.7
Lipidomics at baseline								
Palmitic Acid	~20–25%	23.5	22.9	23.8	19.5	23.2	21	20.4
Stearic Acid	~10–15%	*16.4	*18.9	*16.7	*18	*19.8	*17.1	*17.2
Oleic Acid	~15–25%	19.5	20.5	19.7	16.4	16.6	20	19.3
Linoleic Acid (Omega-6)	~1.3-2.9%	2.1	2.9	1.7	2.1	2.2	2.5	2.9
Arachidonic Acid (Omega-6)	~15–25%	18.2	18.4	20.8	23	21.5	22.1	23.8
EPA (Omega-3)	1-3%	*0.5	*0.5	*0.5	*0.6	*0.5	*0.6	*0.7
DHA (Omega-3)	3-6%	5.7	4.4	5	5.6	5.1	5.4	4.3
DHA+EPA	<4%: Low 4–8%: Moderate >8%: Optimal	6.2	4.9	5.5	6.2	5.6	6	5
Omega-6/Omega-3 Ratio	3.5 -5.5	5.3	*6.4	*5.9	*5.6	*6	*5.8	*7.3

Behavioral measures include the CARS-2-ST (total score; cut-off >30 indicating autism), the DASH-II (severity and frequency ratings across psychiatric and neurodevelopmental domains, with no specific cut-off values), the Short Sensory Profile (SSP) (total and subscale scores, with clinical cut-offs indicating sensory processing difficulties), and the Aberrant Behavior Checklist (ABC) with normative ranges for subscales. Biological markers include fecal calprotectin (inflammation), HbA1c (glucose metabolism), creatine kinase (muscle metabolism), C-reactive protein and interleukin-6 (inflammation), vitamin D, and plasma fatty acid composition (lipidomics). Asterisks and bold (*) indicate values falling outside normative ranges or clinical thresholds.

At baseline, all participants scored above the clinical threshold on the CARS-2-ST, confirming moderate to severe ASD symptoms. The cohort showed high inter-individual variability in symptom severity and behavioral profiles. According to DASH-II and ABC assessments, psychiatric comorbidities such as impulse control problems, manic symptoms, and stereotypic behaviors were common. Irritability and hyperactivity emerged as the most pronounced behavioral challenges, with notable variability across participants. Sensory processing abnormalities were identified in all individuals via the SSP, particularly in the tactile and sensory-seeking domains. Biological assessments revealed no signs of gluten sensitivity, but elevated fecal calprotectin in some cases suggested intestinal inflammation. Most participants exhibited suboptimal metabolic and inflammatory profiles, including elevated HbA1c levels. Notably, IL-6 levels were increased in most individuals, indicating a state of systemic inflammation. Severe vitamin D deficiency was detected in a subset of participants. Lipidomic analyses indicated consistent alterations in erythrocyte membrane composition, particularly elevated stearic, arachidonic acid and an imbalanced omega-6/omega-3 ratio, suggestive of a pro-inflammatory state. All individual data and reference thresholds are presented in **Table 1**.

### 3.3 Comparison of ABC scores over time

**Figure 1:**
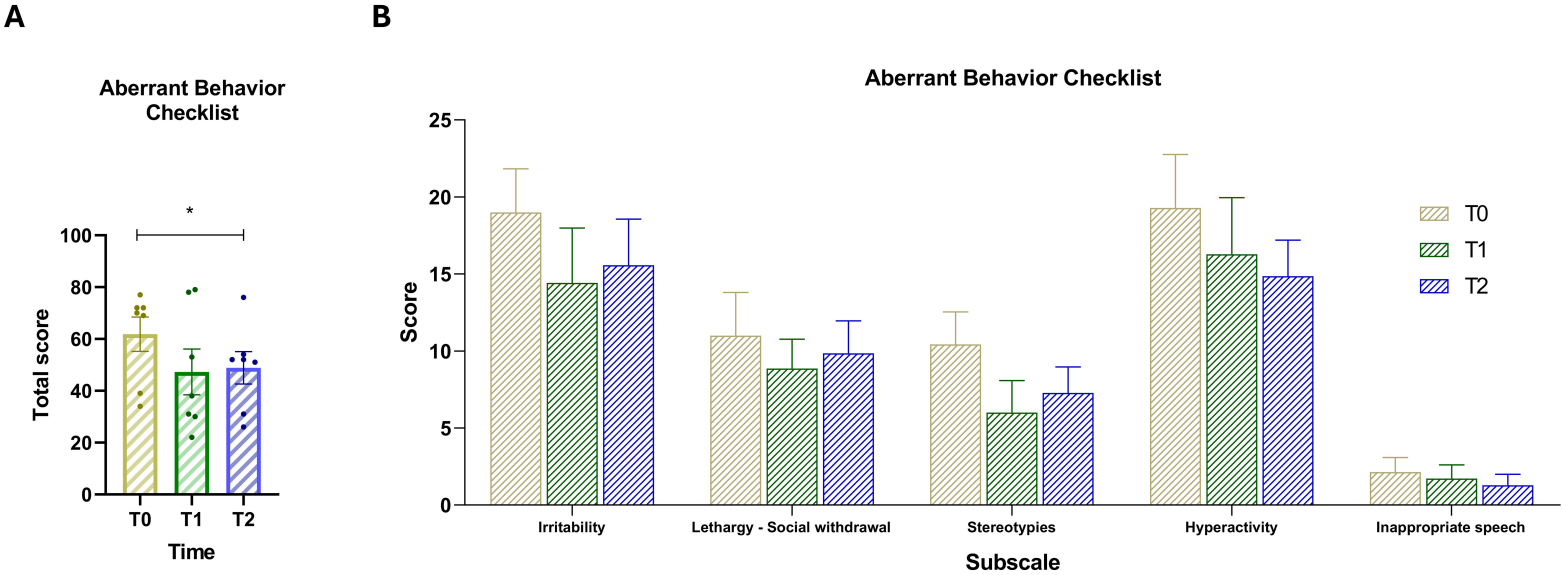
**(A)** Total score of the Aberrant Behavior Checklist (ABC) across the three time points. **(B)** Scores for the five ABC subscales: (1) Irritability, (2) Lethargy/Social withdrawal, (3) Stereotypy, (4) Hyperactivity, and (5) Inappropriate speech. Vertical bars represent the standard error of the mean (SEM). Horizontal lines indicate statistically significant differences between conditions based on *post-hoc* Dunnett’s tests. Asterisks indicate significance levels: ns, not significant, p < 0.05 (*).

The results of the ABC comparison provide meaningful insights into the changes in autism-related behaviors and symptom severity over time (**Figure 1A**). *Post-hoc* comparisons were conducted on the scale’s total score using Dunnett’s test to control for multiple comparisons within a single family (two comparisons, family-wise alpha = 0.05). Results revealed a significant difference between T0 and T2 (mean difference = 13.00, 95% CI [1.23, 24.77], *p* = .0343). Improvements were observed across all subscales (**Figure 1B**), as scores consistently decreased from the beginning to the end of the intervention period. The overall downward trend suggests that dietary intervention may have contributed to a reduction in these challenging behaviors over time.

### 3.4 Comparison of CARS-2-ST scores over time

**Figure 2:**
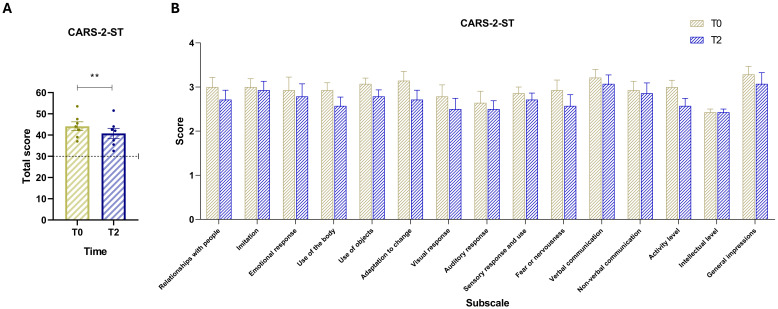
**(A)** CARS-2-ST total scores at baseline (T0, yellow) and post-intervention (T2, blue). The dashed horizontal line indicates the test cut-off score of 30. **(B)** Scores for the 15 CARS items: (1) Relating to people, (2) Imitation, (3) Emotional response, (4) Body use, (5) Use of objects, (6) Adaptation to change, (7) Visual response, (8) Auditory response, (9) Sensory response and use, (10) Fear or nervousness, (11) Verbal communication, (12) Nonverbal communication, (13) Activity level, (14) Intellectual response, and (15) General impressions. Vertical bars represent the standard error of the mean (SEM). Horizontal lines indicate statistically significant differences between conditions based on t-test results. The dashed horizontal line indicates the cut-off for optimal values. Asterisks denote significance levels: ns, not significant, p < 0.01 (**).

The analysis of CARS-2-ST compares T2 values with baseline values (**Figure 2A**). A paired-samples *t*-test revealed a statistically significant decrease in scores between the two time points, *t*(6) = 4.28, *p* = .0052 (two-tailed). The mean difference was –3.36 (SD = 2.08, SEM = 0.78), with a 95% confidence interval ranging from –5.28 to –1.44. The effect size was large, with a partial eta squared (*R²*) of 0.75. This result indicates a general improvement in autism-related behaviors. At T2, all CARS-2-ST subscale scores showed a downward trend compared to baseline (T0), indicating overall improvements across multiple behavioral domains. Importantly, despite these improvements, all participants remained above the CARS-2-ST cut-off score, continuing to exhibit at least moderate levels of autism-related symptoms at T2. This indicates that while the intervention may have reduced symptom severity, it did not lead to a shift below diagnostic thresholds.

### 3.5 Comparison of sensory processing between baseline and T2

**Figure 3:**
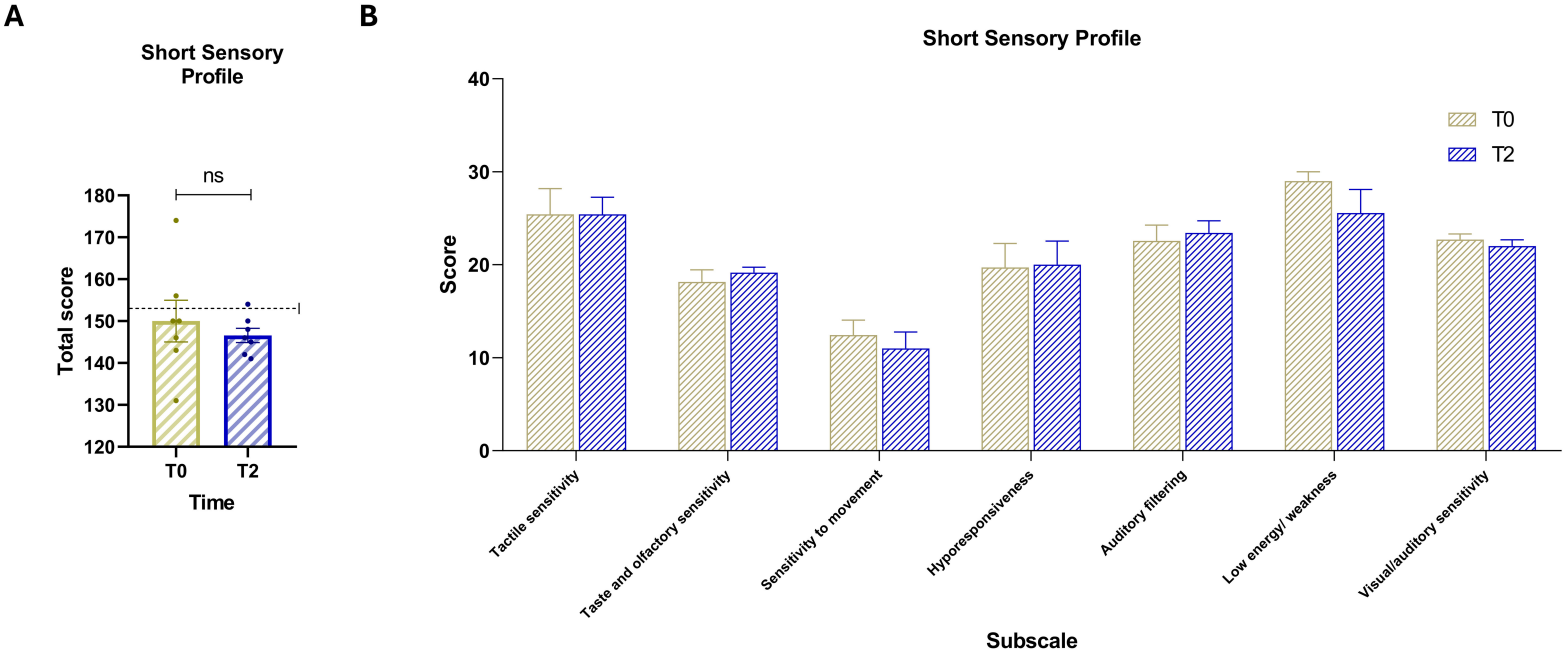
**(A)** Total scores of the Short Sensory Profile (SSP) at baseline (T0, yellow) and post-intervention (T2, blue). The dashed horizontal line indicates the test cut-off score of 154. **(B)** Scores for the seven sensory domain subscales: (1) Tactile sensitivity, (2) Taste and smell sensitivity, (3) Movement sensitivity, (4) Underresponsiveness/seeking behavior, (5) Auditory filtering, (6) Low energy/weakness, and (7) Visual/auditory sensitivity. Vertical bars represent the standard error of the mean (SEM). Horizontal lines indicate statistically significant differences between conditions based on t-test results. The dashed horizontal line indicates the cut-off for optimal values. Asterisks denote significance levels: ns, not significant.

Comparison of SSP scores between baseline and T2 (**Figure 3**) showed a general trend of reduction or stability. The paired-samples t-test comparing total SSP scores between baseline and T2 (**Figure 3A**) did not yield a statistically significant difference, t (6) = 0.85, p = .4293 (two-tailed). The mean difference was –3.43 (SD = 10.71, SEM = 4.05), with a 95% confidence interval ranging from –13.33 to 6.47. The partial eta squared was 0.11, and the pairing correlation was r = 0.66 (p = .0529, one-tailed), not reaching statistical significance. Despite the lack of statistical significance, all participants at T2 had total SSP scores below the clinical cut-off of 154, indicating scores within the normative range for sensory processing. At the subscale level, score trends were either decreasing or remained stable between baseline and T2 (**Figure 3B**).

### 3.6 Changes in DASH-II items over time

**Figure 4:**
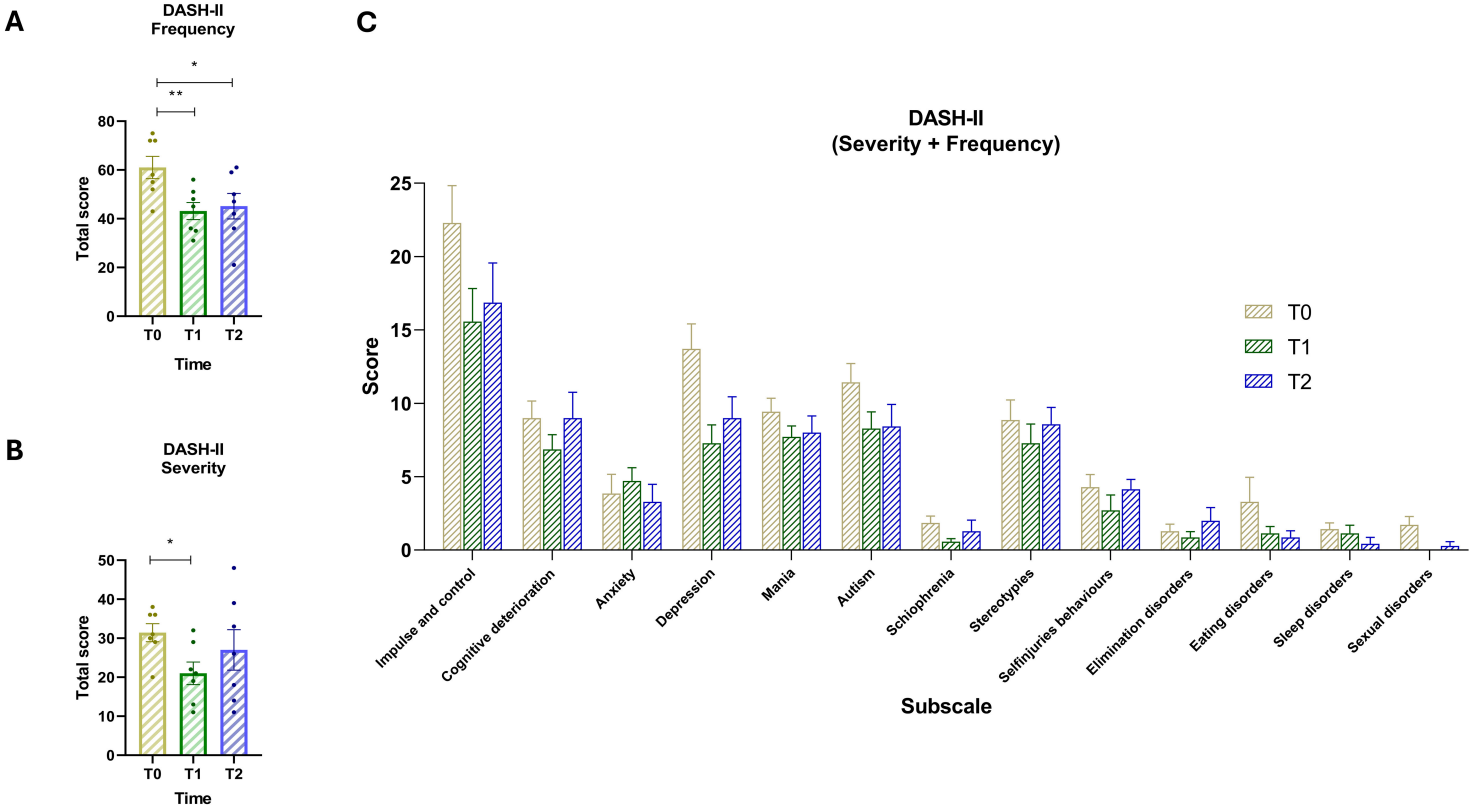
**(A)** Total scores of DASH-II Frequency for time points T0 (yellow) T1 (green) and T2 (blue). **(B)** Total scores of DASH-II Severity scale across the three time points. **(C)** Score of severity and frequency for the 13 subscales: (1) Impulse and control, (2) Cognitive deterioration, (3) Anxiety, (4) Depression, (5) Mania, (6) Autism, (7) Schizophrenia, (8) Stereotypies, (9) Self-injuries behaviors, (10) Elimination disorders, (11) Eating disorders, (12) Sleep disorders, and (13) Sexual disorders. Vertical bars represent the standard error of the mean (SEM). Horizontal lines indicate statistically significant differences between conditions based on *post-hoc* Dunnett’s tests. Asterisks denote significance levels: ns, not significant, p < 0.05 (*), p < 0.01 (**).

The analysis of DASH-II items over two time intervals—Baseline to T1 and T1 to T2—reveals notable patterns in both frequency and severity (**Figure 4A**). A repeated-measures ANOVA revealed a statistically significant effect of time on frequency scores, F(1.475, 8.847) = 11.10, p = .0057, with Greenhouse-Geisser correction (ϵ = 0.7373), and an associated R² of 0.65. The pairing was also statistically significant, F(6, 12) = 5.05, p = .0084, R² = 0.47, indicating consistent within-subject differences. *Post-hoc* Dunnett’s tests showed a significant reduction in behavioral frequency from T0 to T1 (mean difference = 17.86, 95% CI [9.61, 26.11], p = .0015), and from T0 to T2 (mean difference = 15.86, 95% CI [3.95, 27.77], p = .0157). *Post-hoc* analysis on the severity scale (**Figure 4B**) indicated a significant reduction in severity scores between T0 and T1 (mean difference = 10.43, 95% CI [2.05, 18.81], p = .0210), while the difference between T0 and T2 was not statistically significant (mean difference = 4.43, 95% CI [–7.96, 16.81], p = .5247). Comparing T0 and T2 (**Figure 4B**), most symptoms show an overall reduction in both frequency and severity, despite some fluctuations between T1 and T2. Self-Injury decreases slightly from 4.29 at baseline to 4.14 at T2, after a sharper drop at T1 (2.71), indicating a lasting but partial improvement. Impulse and Control behaviors decline from 22.29 to 16.86, reflecting a sustained reduction over time. Cognitive Deterioration remains stable (9.00 at both T0 and T2), suggesting no significant change in this domain. Anxiety decreases from 3.86 to 3.29, showing modest improvement. Depression initially drops from 13.71 to 7.29 at T1 but rises back to 9.00 at T2, remaining lower than baseline. Mania shows a slight reduction from 9.43 to 8.00. ASD symptoms decrease from 11.43 to 8.43, supporting gradual improvement. Schizophrenia-related symptoms reduced from 1.86 to 1.29. Stereotypies, despite increasing from 7.29 to 8.57 between T1 and T2, remain below baseline levels (8.86). Elimination disorders increased from 1.29 to 2.00, representing a worsening. Eating disorders improved from 3.29 to 0.86. Sleep disorders decline from 1.43 to 0.43, and Sexual disorders nearly disappear, going from 1.71 to 0.29.

### 3.7 Changes in biomarkers over time

**Figure 5:**
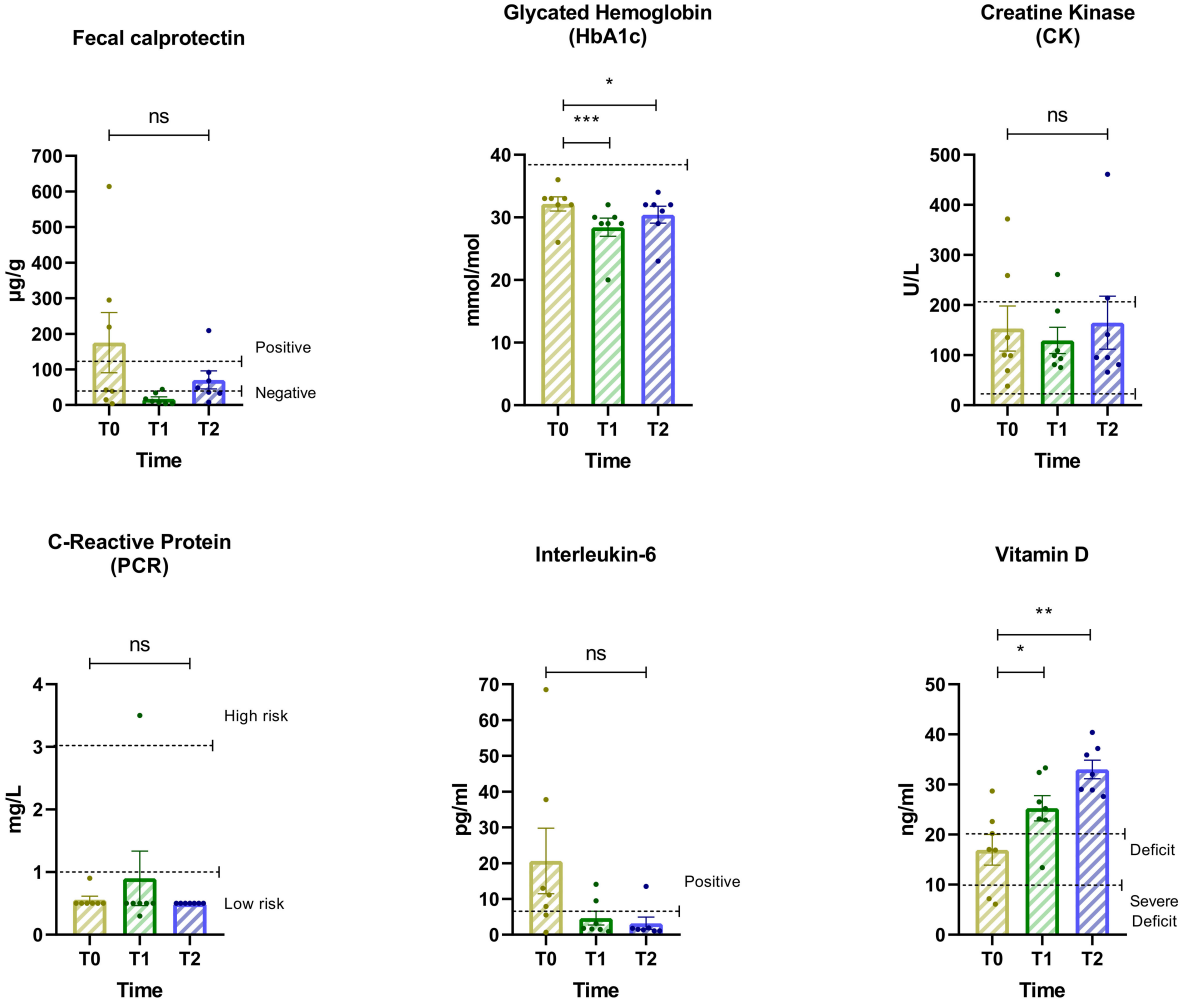
Changes in biomarker levels across three assessment intervals. Biomarkers include: Hemoglobin glycate, Creatine kinase (CK), C-reactive protein (CRP), Interleukin-6, Basal insulin, Vitamin D, and Fecal calprotectin. Horizontal lines indicate statistically significant differences between conditions based on *post-hoc* Dunnett’s tests. The dashed horizontal line indicates the cut-off for optimal values. Asterisks denote significance levels: ns, not significant, p < 0.05 (*), p < 0.01 (**), p < 0.001 (***).

This analysis examines changes in key biomarkers across the three time periods (**Figure 5**). The findings highlight dynamic shifts in biomarker levels, indicating potential metabolic and inflammatory responses to dietary and psychiatric interventions. IL-6 and Fecal calprotectin exhibited non-significant reductions from Baseline to T2 but went back into the accepted range suggesting improvements in inflammatory balance and neurochemical stability. Oxidative Stress Indicator (PCR) showed an increase from T0 to T1, possibly reflecting an adaptive response to initial dietary changes. However, a stabilization trend emerged from T1 to T2, indicating a potential normalization over time. Metabolic Dysregulation (Vitamin D) showed a significant increase from T0 to T1 and T0 to T2. The ANOVA yielded F = 27.84, p = 0.0004. Significant differences were also observed in Insulin Sensitivity (HbA1c) across time points. The repeated measures ANOVA showed a robust effect with F = 38.10, p < 0.0001. *Post-hoc* comparisons using Dunnett’s test revealed significant increases in HbA1c from baseline (T0) to T1, with a mean difference of 3.71 (95% CI: 2.51 to 4.92; p = 0.0002), and a smaller but still significant increase from T0 to T2, with a mean difference of 1.71 (95% CI: 0.51 to 2.92; p = 0.0116). Minor fluctuations were observed in Lipid Profile (CK), with moderate reductions in the first phase followed by stabilization.

### 3.8 Changes in lipidomic indices over time

**Figure 6:**
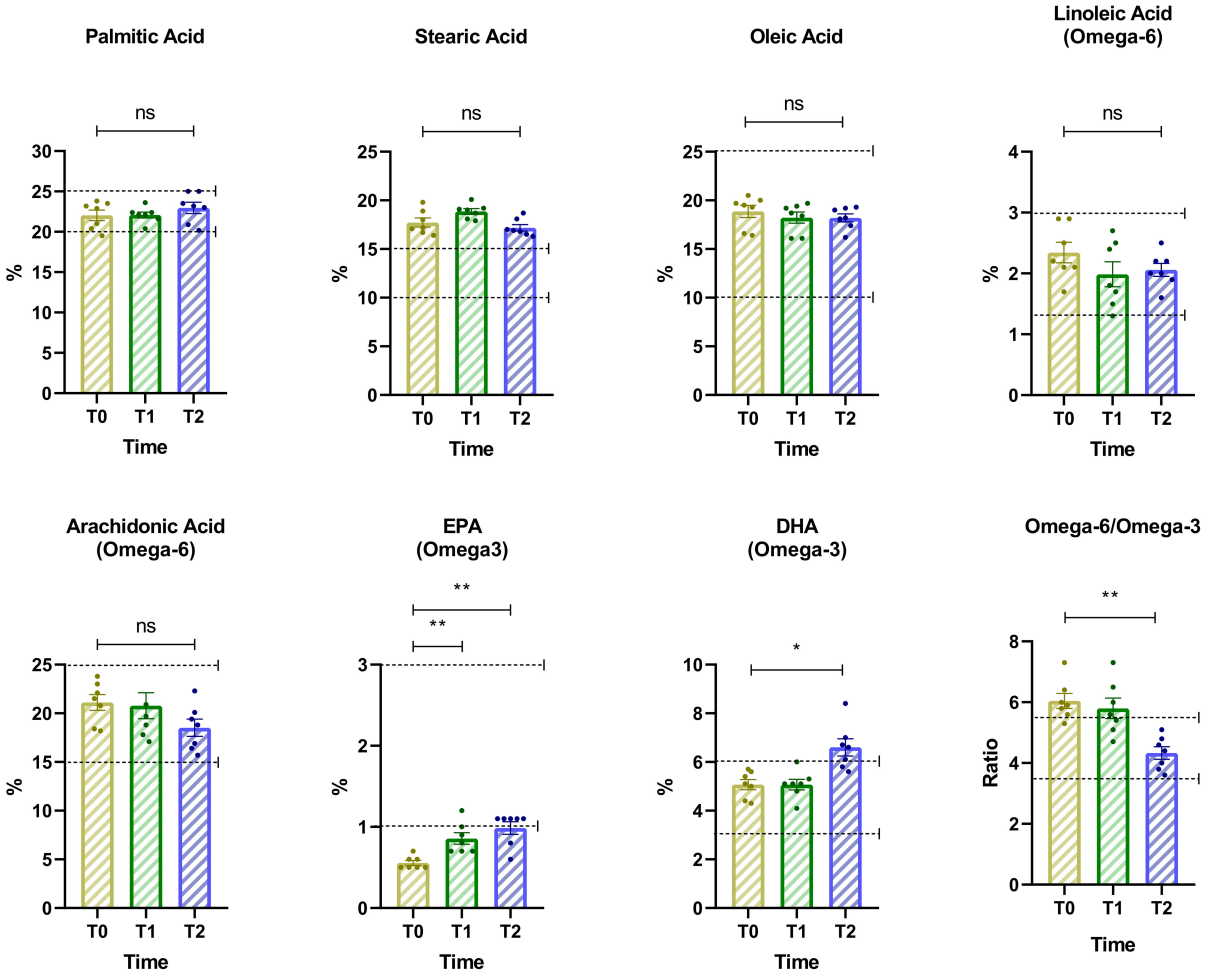
Changes in key lipidomic indices associated with fatty acid metabolism and lipid balance across three time points. The dashed horizontal line indicates the cut-off for optimal values. Asterisks denote significance levels: ns, not significant, p < 0.05 (*), p < 0.01 (**).

This analysis examines percentage changes in key lipidomic indices associated with fatty acid metabolism and lipid balance across the three time points (**Figure 6**). The results highlight significant shifts in lipid profiles, which may reflect metabolic adaptations to dietary or psychiatric interventions. DHA and EPA levels showed a moderate increase from Baseline (T0) to T1, followed by a more pronounced increase from T1 to T2. For DHA, repeated measures ANOVA indicated a significant effect over time (F = 13.06, p = 0.0037, Geisser-Greenhouse epsilon = 0.7265, R² = 0.6852). Dunnett’s multiple comparisons test showed a significant increase between T0 and T2 with a mean difference of -1.53 (95% CI: -2.62 to -0.44, p = 0.0124). For EPA, the ANOVA also revealed a significant effect (F = 15.46, p = 0.0019, Geisser-Greenhouse epsilon = 0.7503, R² = 0.7204). Dunnett’s test indicated significant increases from T0 to T1 (mean difference -0.30, 95% CI: -0.49 to -0.11, p = 0.0067) and from T0 to T2 (mean difference -0.43, 95% CI: -0.62 to -0.23, p = 0.0013).

Similarly, the omega-6 to omega-3 ratio showed a significant reduction over time, consistent with the observed increase in EPA and DHA concentrations. Repeated measures ANOVA indicated a significant time effect (F = 10.93, p = 0.0021, Geisser-Greenhouse epsilon = 0.9837, R² = 0.6456). Dunnett’s multiple comparisons test revealed a statistically significant decrease from T0 to T2 (mean difference = 1.714, 95% CI: 0.6350 to 2.794, p = 0.0070). Across the three time points, levels of palmitic, stearic, and oleic acids remained within optimal ranges, showing only minor fluctuations without statistically significant changes.

### 3.9 Correlation between biomarkers, lipidomic indices, and behavior

**Figure 7:**
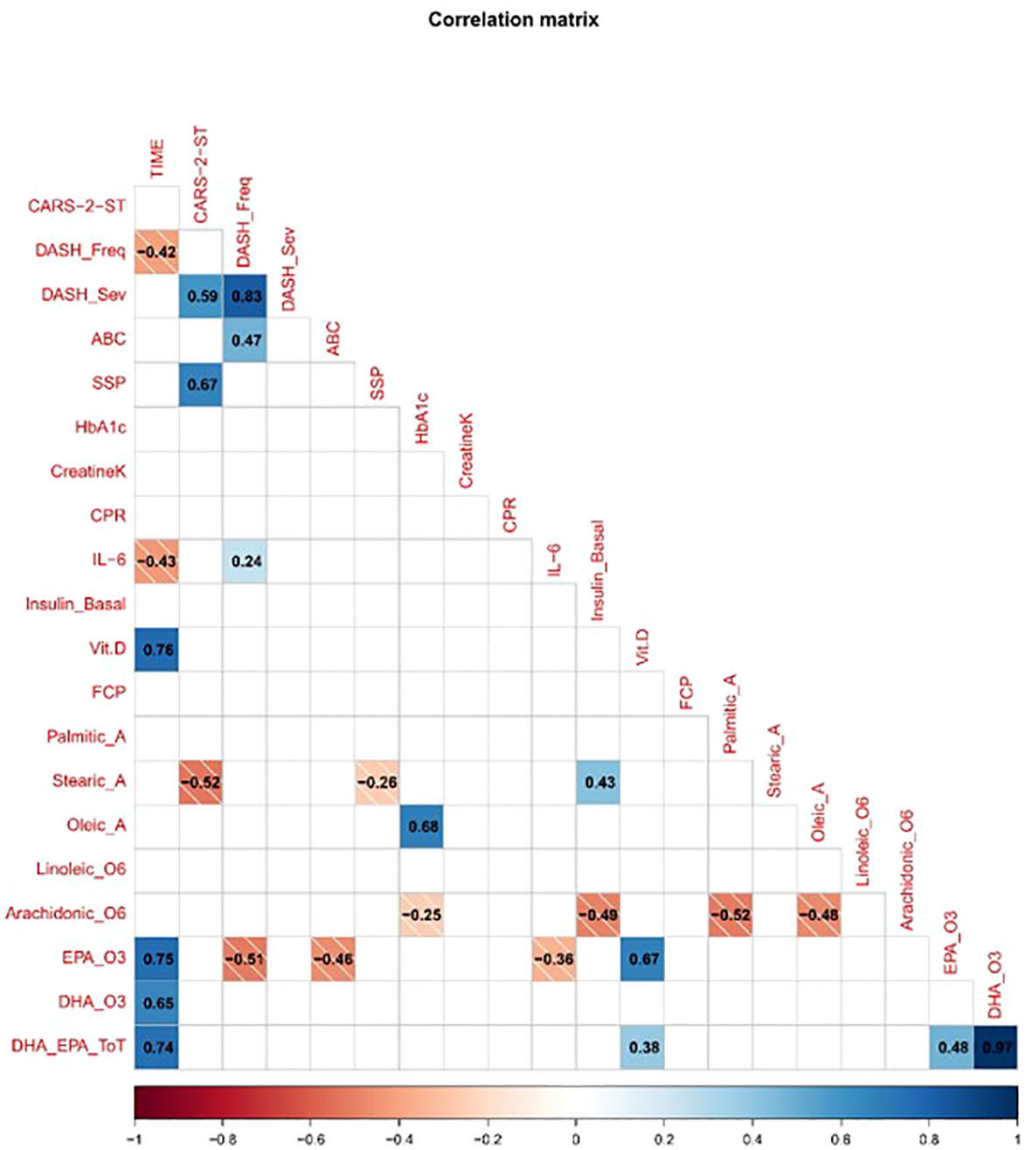
Correlation matrix of clinical, behavioral, and biological variables. The matrix displays pairwise Pearson correlation coefficients, with blue indicating positive correlations and red indicating negative correlations. The strength of correlation is represented by both color intensity and numerical values within each cell. Only significant correlations (p < 0.05) are shown. Variables include CARS-2-ST, DASH-II (frequency and severity), ABC subscales, sensory profile domains, and various biomarkers (e.g., CRP, Interleukin-6, Vitamin D, Fecal Calprotectin, Omega3).

The correlation matrix presented in **Figure 7** shows relationships between neurobiological indices and behavioral measures across the three time points. IL-6 and PCR displayed moderate to strong positive correlations with DASH-II frequency and severity scores. Fecal calprotectin levels correlated positively with ABC scores. HbA1c and fasting insulin levels correlated positively with CARS-2-ST and DASH-II scores. The omega-6 to omega-3 ratio, specifically linoleic acid and arachidonic acid levels, correlated positively with ASD symptom severity and SSP scores. Oleic acid and palmitic acid levels correlated negatively with symptom severity. Vitamin D levels correlated negatively with DASH-II severity scores.

## 4 Discussion

This pilot study represents a significant advance in nutritional psychiatry by applying a biomarker-guided, precision nutrition framework to a population rarely included in clinical research: adults with Level 3 ASD residing in long-term care. Despite severe cognitive, communicative, and behavioral challenges (55, 58), their inclusion demonstrates both the feasibility and ethical necessity of conducting inclusive research.

We designed meals to optimize anti-inflammatory nutrients, emphasizing low-glycemic carbohydrates and omega-3-rich foods like fatty fish, flaxseed, and walnuts, alongside antioxidant-rich fruits and vegetables. At the same time, sugar, ultra-processed foods, and pro-inflammatory seed oils were minimized (59–64). Importantly, the meal plans also took into account the sensory sensitivities and behavioral rigidities common in this population, a critical factor in ensuring long-term adherence (65). A pivotal element of our approach was that meals were co-developed with clinicians, caregivers, and chefs to enhance adherence and acceptability (66, 67), transforming complex nutritional science into tangible, sustainable practice within a residential care context (17, 65, 68, 69).

Our findings indicate that biologically informed nutritional strategies can produce meaningful improvements in both behavior and biochemistry. The ABC scale revealed significant reductions in total and subscale scores from baseline to study end, supporting the idea that dietary modulation can reduce a wide range of externalizing and internalizing behaviors. Similarly, the CARS-2-ST showed a significant decline in autism-related symptoms by study completion, suggesting a strong clinical impact. While all participants remained above diagnostic thresholds, these changes represent meaningful functional gains. These behavioral improvements align with previous findings that diets targeting inflammation and neurochemical stability may quickly influence affective dysregulation and cognitive rigidity (70–72). The DASH-II also confirmed these trends, showing significant early decreases in frequency and severity of challenging behaviors—especially impulse control and self-injury—that were largely maintained at study end. The greatest improvements occurred during the initial intervention phase, indicating some behavioral domains respond rapidly to dietary changes. However, not all trends were uniformly positive. Stereotypies initially declined but increased somewhat between mid- and end-points, though remaining below baseline. This rebound may reflect compensatory mechanisms: as overt externalizing behaviors diminished, internally driven or self-stimulatory behaviors may have become more evident. Alternatively, persistent sensory or arousal regulation challenges might require additional interventions beyond diet (73), Supporting this, the SSP results trended toward normalization but showed no significant change, suggesting sensory symptoms may improve more slowly or be resistant to nutritional modulation. The variability across behavioral domains and time points points to distinct response profiles or biological subtypes within the cohort. Behaviors like impulsivity, irritability, and aggression seem more sensitive to early dietary intervention, whereas stereotypy or depression may need longer or adjunctive therapies (e.g., occupational or sensory integration approaches). This underscores the importance of personalized, biomarker-informed treatments tailored to specific symptoms and neurobiological profiles.

Biomarker data suggest asynchronous physiological adaptation. Early trends in IL-6 and fecal calprotectin may reflect an acute metabolic or immune adjustment common to nutrient or microbiota shifts (74, 75). The significant increase in vitamin D and shifts in DHA and EPA point to improved micronutrient status and lipid signaling, which may enhance neuroplasticity and synaptic function, supporting long-term behavioral gains. The gradual rise in omega-3 fatty acids aligns with known temporal delays in lipidomic remodeling (70). Conversely, the rise in HbA1c adds complexity—it may reflect transient shifts in energy metabolism or hormonal regulation associated with the intervention rather than pathology, emphasizing the need for ongoing metabolic monitoring. Overall, biomarker patterns support a model of coordinated, time-dependent modulation of multiple systems: early inflammatory and lipid changes may precede later metabolic and neuroendocrine shifts, indicating an actively adapting physiological system with partial normalization alongside emerging vulnerabilities.

Despite sharing the Level 3 ASD diagnosis, participants exhibited marked variability in peripheral lipid profiles, especially in omega-6 to omega-3 PUFA ratios. This heterogeneity highlights limitations of categorical diagnoses and reinforces the need for biologically grounded subtyping, consistent with the RDoC framework (76). Importantly, more pro-inflammatory lipid profiles correlated with more severe behavioral symptoms, while higher omega-3 and monounsaturated fatty acid levels associated with better affect regulation, reduced aggression, and improved social engagement. These relationships have strong biological bases: long-chain PUFAs, particularly omega-3 (EPA, DHA) and omega-6 (arachidonic acid), are vital to neuronal membrane integrity, influencing synaptic plasticity, receptor function, membrane fluidity, and neurotransmission (59, 72). Excess omega-6 fatty acids promote neuroinflammation via pro-inflammatory eicosanoids (77), activating microglia and disrupting neuron-glia communication, which can increase stress sensitivity and dysregulate mood and behavior (78, 79). Conversely, omega-3s generate pro-resolving mediators that reduce inflammation and enhance neuronal resilience (80). DHA also supports lipid raft formation (81, 82), critical for receptor and endocannabinoid signaling implicated in ASD sensory and anxiety symptoms (83). PUFA imbalances may disrupt dopamine and serotonin pathways, linked to aggression, rigidity, and mood issues central to severe ASD (84–86). Thus, restoring lipid balance may improve outcomes across multiple neurobiological pathways. While similar lipidomic alterations have been reported in children with ASD or related neuropsychiatric disorders (87–93), to our knowledge, this is the first study designed to implement personalized nutritional interventions in adults with level 3 ASD. Previous high-profile trials in nutritional psychiatry, such as the SMILES trial for major depressive disorder, which demonstrated that adherence to a Mediterranean diet improved depressive symptoms (60), have largely focused on more accessible and verbally fluent populations.

Our findings extend this evidence by demonstrating that individuals with profound neurodevelopmental impairments can also benefit from biologically grounded, nutrition-driven approaches. What makes these findings particularly compelling is not merely the observed outcomes, but the context in which they occurred. Adults with Level 3 ASD typically present with significant challenges that complicate both daily care and participation in clinical research, including behavioral rigidity, limited communication, sensory aversions, and multiple psychiatric comorbidities (94). As a result, research involving this group is often characterized by small sample sizes, limited intervention feasibility, and high attrition rates (95–100). In this study, however, all participants completed the full intervention period with high adherence and no dropouts, despite the diagnostic and behavioral complexity of the group. This not only reinforces the feasibility and robustness of the intervention model but also highlights its strong ecological validity. This study’s success underscores the power of a systems-based, person-centered approach. Our sample size, despite being small, exemplifies the feasibility of delivering intensive, biomarker-informed interventions within a vulnerable population (101). Though lacking neurotypical or clinical control groups—due to ethical and logistical challenges in this setting—we employed a within-subject, intra-cohort design focusing on lipidomic-behavior relationships. This dimensional strategy aligns with transdiagnostic models and supports precision nutrition tailored to individual biology rather than population averages (102).

Overall, this study exhibits several key strengths. The co-designed intervention model—tailored in both content and delivery to meet the specific sensory and behavioral profiles of the target population—was embedded directly within residential care routines, enhancing ecological validity. The multimodal evaluation strategy, integrating behavioral, sensory, biochemical, and lipidomic measures, provided a comprehensive lens on intervention impact. Moreover, the adoption of an intra-individual analytical approach allowed the study to transcend diagnostic categories and engage directly with biologically meaningful dimensions of treatment response. At the same time, the study’s limitations warrant careful consideration. The small sample size reduces statistical power and restricts subgroup analyses. The lack of a control group prevents causal attribution of observed effects to the dietary intervention. Interpretation of some biochemical shifts—such as the increase in HbA1c—remains challenging without extended follow-up. Additionally, the considerable heterogeneity observed in both behavioral and biomarker data underscores the need for biologically stratified designs in future research, ideally incorporating lipid profiles and inflammatory markers to guide participant selection and intervention tailoring (103). Despite these limitations, the results strongly support the feasibility, acceptability, and translational value of precision nutrition strategies in adults with complex neurodevelopmental conditions. Replication in larger, more diverse cohorts is a critical next step, along with integration of additional omics layers—such as microbiome, proteomics, and genomics—to further elucidate mechanisms and refine therapeutic targets. By enabling biologically defined ASD subtypes, future interventions can move toward precision-guided, individualized care that is both scalable and clinically meaningful.

Thus, this study affirms that even the most behaviorally and medically complex individuals can benefit from science-driven, biologically grounded interventions. In doing so, it challenges existing assumptions about feasibility and sets a precedent for inclusive, real-world applications of precision psychiatry. Through this work, a long-marginalized population is not only represented—but empowered to help shape the future of translational science.

## 5 Conclusion and future perspectives

This study provides compelling evidence that precision nutrition—grounded in detailed metabolic and lipidomic profiling—can meaningfully modulate key biological pathways and improve behavioral symptoms in adults with severe ASD. By embracing a personalized, biomarker-driven approach and integrating dietary interventions into real-life settings, we demonstrate a promising new frontier in nutritional psychiatry. These findings not only validate the therapeutic potential of targeted dietary strategies but also pave the way for a paradigm shift toward more individualized, biologically informed treatments for ASD and related neurodevelopmental disorders.

## Data Availability

The raw data supporting the conclusions of this article will be made available by the authors, without undue reservation.
